# The Cost-Effectiveness of the BEAT-TB Regimen for Pre-Extensively Drug-Resistant TB

**DOI:** 10.3390/tropicalmed8080411

**Published:** 2023-08-11

**Authors:** Malaisamy Muniyandi, Paranchi Murugesan Ramesh, William A. Wells, Umesh Alavadi, Suvanand Sahu, Chandrasekaran Padmapriyadarsini

**Affiliations:** 1 ICMR-National Institute for Research in Tuberculosis, Chennai 600031, India; padmapriyadarsi.nic@icmr.gov.in; 2 Government Ottery TB Hospital, Ottery, Chennai 600012, India; pmrmdchest2@gmail.com; 3 United States Agency for International Development (USAID), Washington, DC 20004, USA; wwells@usaid.gov; 4 United States Agency for International Development (USAID), Chanakyapuri, New Delhi, Delhi 110021, India; ualavadi@usaid.gov; 5 Stop TB Partnership Secretariat, 1218 Geneva, Switzerland; sahus@stoptb.org

**Keywords:** tuberculosis, infectious diseases, economic impact, pre-XDR-TB treatment regimen, shorter BEAT-TB India regimen, societal perspective, cost-effective BEAT-TB regimen

## Abstract

Objective: To measure the economic impacts of the longer pre-XDR-TB treatment regimen and the shorter BEAT-TB India regimen. Methods: In the current study, the economic impacts of the current 18-month pre-XDR-TB treatment regimen and the 6–9 month BEAT-TB regimen were evaluated using an economic model via a decision tree analysis from a societal perspective. The incremental costs and quality-adjusted life years (QALYs) gained from the introduction of the BEAT-TB regimen for pre-XDR-TB patients were estimated. Results: For a cohort of 1000 pre-XDR-TB patients, we found that the BEAT-TB India regimen yielded higher undiscounted life years (40,548 vs. 21,009) and more QALYs gained (27,633 vs. 15,812) than the 18-month regimen. The BEAT-TB India regimen was found to be cost-saving, with an incremental cost of USD −128,651 when compared to the 18-month regimen. The current analysis did not consider the possibility of reduced TB recurrence after use of the BEAT-TB regimen, so it might have under-estimated the benefits. Conclusion: As a lower-cost intervention with improved health outcomes, the BEAT-TB India regimen is dominant when compared to the 18-month regimen.

## 1. Introduction

Tuberculosis (TB) is the second-most-common cause of death globally from an infectious disease (WHO, 2021). There are concerns for achieving the UN General Assembly targets of eliminating TB as a global health threat by 2035. The WHO’s annual TB Report (2021) estimates that there were 10 million new TB infections and 1.5 million deaths globally [1,2]. Drug-resistant tuberculosis (TB) is a major global health risk, driving the ongoing TB epidemic and increasing the morbidity and mortality of TB worldwide (WHO TB report 2021). Incomplete and insufficient treatment regimens can lead to antimicrobial resistance. Earlier detection requires access to care and rapid diagnostic tools, which may be limited in many areas. Once multidrug-resistant TB (MDR-TB) treatment is initiated, adherence and tolerability may be a challenge. Recent evidence suggests that MDR-TB is an important contributor of Post-TB Lung Disease (PTLD), which is responsible for disability and suffering, often requiring rehabilitation [3].

Globally, multidrug-resistant tuberculosis (MDR-TB) continues to be a major public health concern. As per the Global TB Report 2022, there were an estimated 450,000 new cases (95% UI: 399,000–501,000) of MDR/RR-TB in 2021, with India alone contributing 26% of global cases. Also, in 2021, 2021 the estimated global proportion of MDR-TB that is pre-extensively drug-resistant TB (pre-XDR-TB, i.e., MDR/RR-TB with additional resistance to any fluoroquinolone) was 20% (95% CI: 16–26%) [2]. The India TB Report 2023 reported that in 2022, out of 63,801 MDR/RR-TB cases diagnosed, only 23,846 (37%) had valid drug susceptibility test results available for fluoroquinolone, 12,002 of which were pre-XDR-TB. Thus, less than 50% of MDR-TB patients had a drug susceptibility test available for fluoroquinolones and more than 50% of those tested for fluoroquinolones showed pre-XDR-TB [4].

As we wait for the WHO-recommended Bedaquiline–Pretomanid–Linezolid (BPaL) regimen for pre-XDR-TB to be rolled out in India, patients with pulmonary pre-XDR-TB have been treated with the all-oral, longer, 18–20-month regimen of six drugs (Levofloxacin, Linezolid, Clofazimine, Cycloserine, and Bedaquiline) [5]. Though we had shown a high treatment success rate with the BEAT-TB India regimen (6–9 months of Bedaquiline (Bdq), Delamanid (Dlm), Clofazimine (Cfz), and Linezolid (Lzd)) [6], it has yet to be introduced into the national TB elimination programme (NTEP). Here, we aim to evaluate the economic impacts of the longer pre-XDR-TB treatment regimen and shorter BEAT-TB India regimen from a societal perspective, using an economic model via a decision tree analysis.

## 2. Materials and Methods

The objective of this study was to assess and compare the economic impacts of two different treatment regimens for pre-XDR-TB. Specifically, the study focused on comparing the economic outcomes of the 18-month pre-XDR-TB treatment regimen with the BEAT-TB India regimen. To provide a comprehensive analysis, the comparison was conducted by considering a hypothetical cohort consisting of 1000 pre-XDR-TB patients who received their healthcare services in public health facilities located in India.

### 2.1. Intervention and Comparator

We considered the BEAT-TB India regimen as an intervention (Table 1). It was compared with India’s current 18-month regimen for pre-XDR-TB of 18–20 months of Levofloxacin (Lfx), Linezolid (Lzd), Clofazimine (Cfz), and Cycloserine (Cs), plus 6 months or more of Bedaquiline (Bdq), as outlined in the NTEP’s national programmatic management of drug-resistant TB guidelines 2021 [5], which were also used as the comparator arm in the BEAT-TB India study [6]. 

### 2.2. Time Horizon

This study considered the entire treatment period to analyse the costs and outcomes of the BEAT-TB regimen and the current 18–20-month regimen for pre-XDR-TB. Only adult patients were included, with an average age of 32 years and a life expectancy of 44 additional years [6]. We used India’s standard life table to calculate life expectancy and all-cause mortality based on the average age of the cohort. By considering these factors, we aimed to provide an assessment of the economic impacts and effectiveness of the BEAT-TB India regimen and the 18–20-month regimen for pre-XDR-TB.

### 2.3. Model Description

**Figure 1:**
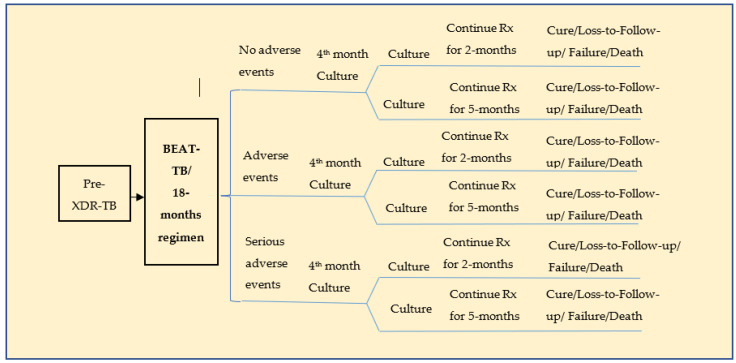
Decision tree for pre-XDR-TB treatment.

A standard hypothetical cohort consisting of 1000 pre-XDR-TB patients with an average age of 32 (range: 20–44) years was considered [6]. This model used only the patients who accessed the public health facilities fortnightly for medication. The patients’ treatment outcomes were classified as cure, lost to follow-up, failure, and death for both the BEAT-TB and the 18-month regimen [6]. The life years (LYs) and quality adjusted life years (QALYs) gained by patients treated in both the regimens were considered the model’s outcomes. Adverse drug reactions (ADRs) attributable to the treatment regimens were also considered. A decision tree model (Figure 1) was constructed based on the proposed and current strategies. Both the BEAT-TB regimen and the current 18-month regimen were modelled as two parallel branches based on the probabilities associated with the treatment outcomes. A patient who underwent TB treatment was classified as having no adverse drug reactions, adverse drug reactions or severe adverse drug reactions for each of these strategies. A Microsoft Excel spreadsheet was used to carry out the analysis.

Table 2 provides the operational definitions of cure, lost to follow-up, treatment failure, death, ADR, and culture conversion. These are the standard definitions that were used in the clinical trials and that are provided in the Indian national programmatic guidelines for tuberculosis. 

### 2.4. Model Input Parameters

The model included key input parameters such as age-specific life expectancy and all-cause mortality [7]. A cohort began with an average age of 32 years (range: 20 to 44 years) for TB patients, and an additional life expectancy of 44 years at age 32 was considered [4]. The clinical outcomes of the BEAT-TB regimen were gathered from the trial: a single-arm cohort study conducted in India [6]. The clinical outcomes for the 18-month regimen were collected from an observational study [8]. The input parameter table (Table 3) also includes the distributions of the input parameters to account for the variability in the inputs. 

The beta distribution was used for the culture conversion outcome, treatment outcome, and the ADRs of the 18–month regimen and BEAT-TB regimen, as they each have a continuous probability distribution, which is used to model probabilities in which the outcome can take any value between 0 and 1. All the costs follow a gamma distribution, as this is a continuous probability distribution and is suitable for modelling non-negative data. 

A beta distribution is characterized by two shape parameters, α and β, which control its shape. To calculate the shape parameters for the beta distribution, we used the following formula: β = ((1 − mean)/variance − 1/mean) × mean^2^ and α = β × ((1/mean) − 1). 

The gamma distribution has two parameters, a shape parameter (k) and a scale parameter (θ), which are used for positive, continuous data. In our cost-effectiveness model, we derived these shape and scale parameters using the standard error and mean. To calculate the standard error, the upper and lower limits were used with the following formula: standard error (SE) = (upper limit − lower limit)/(2 × Z), where Z is the z-score for the 95% confidence level (1.96). The gamma distribution scale and shape parameters (k and θ) were then calculated using the mean and coefficient of variation (CV), with the formulas: (1) CV = standard error/mean; (2) k = (mean/CV)^2^; and (3) θ = mean/k. 

The sample values were then randomly generated using the calculated shape and scale parameter values for each input parameter. These values were used in a probability sensitivity analysis in a Monte Carlo simulation, which was used to check the uncertainty in the model. 

### 2.5. Cost Data

This cost-utility model was performed primarily from a societal perspective which included health system costs and patient costs (i.e., the costs incurred by the individual who accesses treatment services for TB). 

The health system costs comprised the costs of medication [9,10], investigation [11], the human resources used, and hospitalization for ADRs [6,12]. Among them, the medication costs for both regimens were collected from the India mart and Med India websites, whereas staff incentives were collected from NTEP reports [13].

For patients, their out-of-pocket expenditure includes the costs incurred for food and travel as direct costs and the loss of income due to work absenteeism as an indirect cost during treatment. These were collected form the estimates of the Rajan Babu Institute of Pulmonary Medicine and Tuberculosis [14]. Since the regimens have different durations, all these costs were estimated separately for each regimen for the entire course of each respective treatment in terms of Indian rupees.

### 2.6. Effectiveness Data

The quality-of-life scores for cured TB patients were sourced from an Indian study that used a 36-item short form survey (SF-36) [15], whereas for lost to follow-up and failure patients, we utilized scores published from Nigeria [16]. The utility value of well-being was measured on a scale of 0 to 1 in which a score of zero represents death and a score of one indicates perfect health. 

### 2.7. Data Analysis

The current study was evaluated using an economic model via a decision tree analysis. We planned to use an incremental cost/effectiveness ratio (ICER) as an indicator to find the most cost-effective regimen for treating pre-XDR TB. We estimated the incremental cost, QALYs, and life years gained for a total of 1000 patients for both the 18-month treatment regimen and the 6–9-month BEAT-TB regimen. We also performed a probability sensitivity analysis (PSA) and a one-way sensitivity analysis (OWSA) to account for the uncertainty in the model and found that the results were robust to such analyses. Throughout these analyses, the results showed that BEAT-TB is a cost-saving regimen with improved clinical outcomes compared to the 18-month pre-XDR-TB treatment regimen, i.e., a dominant strategy.

### 2.8. Model Outcome Parameters 

The outcomes of the model are expressed in terms of QALYs, LYs, and the overall cost incurred per patient for both the 18-month and the BEAT-TB regimens. 

### 2.9. Study Oversight 

Since this modelling was performed based on secondary data that are freely available from the published literature, the study did not require Institutional Ethics Committee approval. The researchers conducted the study following good reporting practices from the published standard guidelines for conducting and reporting an economic evaluation survey (CHEERS) statement. 

## 3. Results

### 3.1. Base Case Analysis 

The base case analysis for the 1000-patient cohort showed that the total undiscounted costs incurred by both the health system and by patients for the BEAT-TB regimen and the current 18-month regimen were INR 64.9 million (USD 865,000) and INR 75.5 million (USD 1,000,000), respectively. The various health system costs for the BEAT-TB treatment and current 18-month regimen were (1) drugs, INR 51.7 million and INR 43 million; (2) human resources, INR 2 million and INR 5 million; (3) investigation cost, INR 5.9 million and INR 10.8 million; and (4) hospitalization for ADR, INR 0.74 million and INR 1.2 million, respectively. With respect to patient costs for the BEAT-TB treatment and the current 18-month regimen, the costs were (1) direct, non-medical costs for food, INR 3 million and INR 9.5 million; and (2) travel, INR 1.5 million and INR 6 million, respectively. Overall, patient costs for the BEAT-TB and 18-month regimens were INR 4.5 million and INR 15.5 million, respectively. 

### 3.2. Cost and Incremental Effectiveness

For a cohort of 1000 pre-XDR TB patients, we found that the BEAT-TB India regimen yielded higher undiscounted life years (40,548 vs. 21,009) than the 18-month regimen. The QALYs gained as a result of the BEAT-TB India regimen and the 18-month regimen were 27,633 and 15,812, respectively. The BEAT-TB India regimen was found to be cost-saving, with an incremental cost of USD −128,651 (i.e., a savings of USD 128,651) for a 1000 pre-XDR TB patient cohort when compared to the 18-month regimen (Table 4).

As a lower-cost intervention with improved health outcomes, the BEAT-TB India regimen is therefore dominant when compared to the 18-month regimen. These advantages in terms of treatment cost (including both clinical and patient costs), life years gained, and QALYs gained are beneficial for both the patient as well as the health system. Within these calculations, the BEAT-TB India regimen reduces the cost for travel and hospitalization for the management of adverse drug reactions. In total, such evidence could help to accelerate regimen-based decisions relating to pre-XDR TB and thus realize savings and better patient outcomes earlier.

## 4. Discussion

As of March 2023, the treatment of pre-XDR TB in India as per PMDT guidelines is the longer oral regimen of twenty months with bedaquiline (for 6-months), levofloxacin, linezolid, clofazimine, and cycloserine, with the linezolid dose tapered after the initial 6–8 months of treatment [3]. Though, at 69%, the treatment success rate of the 2020 cohort has increased from the success rates of previous cohorts that were on the injectable-containing longer regimen, the current regimen still has a high death rate (16%) and lost to follow-up rate (8%) and a significant incidence of adverse events. In the meantime, we reported the efficacy of an all-oral, shorter regimen of bedaquiline with delamanid and other repurposed drugs under the BEAT-TB India study [6]. The BEAT-TB India study was a prospective, open-label, single-arm cohort study that was conducted at five sites in India where patients with pulmonary pre-XDR TB received 6–9 months of bedaquiline, delamanid, Linezolid, and clofazimine, were followed for 18 months post treatment, and showed a sustained treatment success of >91% at a 6-month post-treatment follow-up [6].

Our model estimates that, if introduced, this 6–9-month BEAT-TB India regimen would be dominant in terms of the number of QALYs gained and the LYs saved when compared to the 18-month pre-XDR-TB treatment regimen. This model was evaluated using an economic model via a decision tree analysis from a societal perspective. For a cohort of 1000 pre-XDR-TB patients, we found that the BEAT-TB India regimen yielded higher undiscounted life years (40,548 vs. 21,009) and more QALYs gained (27,633 vs. 15,812) than the 18-month regimen. The BEAT-TB India regimen was found to be cost-saving, with an incremental cost of USD −128,651 when compared to the 18-month regimen.

In the treatment of DR-TB, patient-friendly regimens are being prioritised to improve treatment outcomes. Regimens with bedaquiline and delamanid, plus one or two additional drugs, have shown higher culture conversions with better treatment outcomes and fewer adverse events, including cardiac events, in patients with pre-XDR-TB [6,17,18,19]. Studies have indicated that despite including two new drugs like pretomanid and bedaquiline, the BPaL regimen is also a less-expensive and cost-saving alternative when compared to conventional regimens for patients with XDR-TB [20,21]. The BEAT-TB regimen can also be considered in patients with pre-XDR-TB, especially when pretomanid is contraindicated or unavailable in the country. Although the cost of delamanid is currently high, which is linked mainly to its small volume of consumption, the price can be potentially reduced by increased procurement volumes and price negotiations, which would further add to the savings outlined in the current analysis. 

## 5. Limitations

This study was evaluated from a societal perspective, including both the patient and provider perspectives. This model only included patients who accessed the public health facilities fortnightly for medication. Further, we did not consider the lifetime benefits of this regimen, so if the BEAT-TB regimen reduced the frequency of recurrent disease, the current analysis might have under-estimated the benefits. In addition, we were not able to compare the other shorter regimens, like BPaL and BPaLM, due to non-availability of India-specific data. Future studies on the cost-effectiveness of shorter regimens in treating patients with pre-XDR-TB could include such regimens in their scope. In addition, we concluded that the BEAT-TB India regimen is a dominant regimen when compared to the 18-month regimen, using input data from two distinct clinical sources. In theory, these findings could be further validated in the future if there is a clinical trial in which both regimens are tested head-to-head, although given the superior outcomes of the shorter regimen, such a trial design is unlikely to be implemented.

## 6. Conclusions

We set out to evaluate the economic impacts of the longer pre-XDR-TB treatment regimen and shorter BEAT-TB India regimen. We found that as a lower-cost intervention with improved health outcomes, the BEAT-TB India regimen is dominant when compared to the 18-month regimen. The BEAT-TB India regimen offers a shorter treatment duration compared to the 18-month regimen. The shorter treatment duration can lead to increased treatment adherence and a reduced burden on patients and can potentially lower the healthcare costs associated with extended treatment periods. If the BEAT-TB India regimen has the potential to be scaled up and implemented more widely across the healthcare system, this regimen could have a significant impact on reducing the overall burden of pre-XDR-TB.

## Figures and Tables

**Table 1:** Treatment intervention for adult, new, smear-positive, drug-sensitive pulmonary TB.

Strategies	Drugs	Regimen	Duration	Population
Current strategy	Levofloxacin (Lfx) Linezolid (Lzd) Clofazimine (Cfz) Cycloserine (Cs) Bedaquiline (Bdq)	(18–20) Lfx Bdq (6 month or longer) Lzd Cfz Cs	18 months	Adult pre-XDR smear-positive pulmonary TB
Proposed Strategy BEAT	Bedaquiline (Bdq) Delamanid (Dlm) Clofazimine (Cfz) Linezolid (Lzd)	(6–9) Bdq Dlm Cfz Lzd	6 months	Adult pre-XDR smear-positive pulmonary TB

**Table 2:** Definition for treatment outcome for adult, new, smear-positive, drug-sensitive pulmonary TB.

Treatment Outcomes	Definition
Cure	A pulmonary TB patient with bacteriologically confirmed TB at the beginning of treatment who completed treatment as recommended by the national policy, with evidence of bacteriological response and no evidence of failure.
Lost to follow-up	A patient who did not start treatment or whose treatment was interrupted for 2 consecutive months or more.
Treatment failure	A patient whose treatment regimen needed to be terminated or permanently changed to a new regimen or treatment strategy
Death	A patient who died before starting treatment or during the course of treatment.
Adverse drug reaction (ADR)	Patients who developed adverse drug reactions attributable to the drugs in the treatment regimen. The ADRs in this study included only cases that were moderate and severe and that required hospitalization.
Culture conversion	Sputum culture conversion was defined as two consecutive bacteriological improvement cultures at the end of the fourth month and the sixth month.

**Table 3:** Input parameters used for the cost-effectiveness analysis of the 6-month BEAT-TB regimen compared to the 18-month standard pre-XDR regimen.

	Input Parameters	Base Case	Lower	Upper	Distribution	Source
Demographic	Average age of TB patient (years)	32 (20–44)	26	38	NA	6
	Cohort population (n)	1000	-	-	NA	-
	Additional years of life expectancy at age 32 years (years)	43.7	35	52	NA	7
	All-cause mortality (per year)	0.009	0.0072	0.0108	Beta	7
Culture conversion (probability)	6-month regimen (BEAT-TB)	0.85	0.68	1	Beta	6
	18-month regimen	0.44	0.35	0.53	Beta	8
Treatment outcome of 6-month regimen (BEAT-TB) (probability)	Mortality due to TB	0.02	0.016	0.024	Beta	6
	Failure	0.01	0.008	0.012	Beta	6
	Cure	0.85	0.68	1	Beta	6
	Lost to follow-up	0.12	0.096	0.144	Beta	6
ADR (BEAT-TB) (probability)	No ADR	0.04	0.032	0.048	Beta	6
	ADR	0.82	0.656	0.984	Beta	6
	Serious ADR	0.15	0.12	0.18	Beta	6
Treatment outcome of 18-month regimen (probability)	Mortality due to TB	0.52	0.41	0.62	Beta	8
	Failure	0.19	0.15	0.22	Beta	8
	Cure	0.26	0.21	0.31	Beta	8
	Lost to follow-up	0.0357	0.0285	0.0428	Beta	8
ADR (18-month regimen) (probability)	No ADR	0.24	0.19	0.28	Beta	8
	ADR	0.52	0.41	0.62	Beta	8
	Serious ADR	0.24	0.19	0.28	Beta	8
Quality of life score	Treatment failure	0.62	0.50	0.74	Beta	15
	Cure	0.87	0.70	1	Beta	14
	Lost to follow-up	0.62	0.50	0.74	Beta	15
Cost for 6-month regimen (BEAT-TB) (INR)	Drugs	51,777	41,422	62,132	Gamma	9, 10
	Chest X-ray	64	51	77	Gamma	11
	Sputum Smear (ZN)	70	56	84	Gamma	11
	Culture (MGIT)	2970	2376	3564	Gamma	11
	CBC	1271	1017	1525	Gamma	5
	ECG	1495	1196	1794	Gamma	5
	Staff incentives	2000	1600	2400	Gamma	13
	Patient incentives (NPY)	3000	2400	3600	Gamma	4
	Travel—patient	1500	1200	1800	Gamma	14
Cost for 18-month regimen (INR)	Drugs	43,013	34,410	51,616	Gamma	9, 10
	Chest X-ray	128	102	154	Gamma	11
	Sputum Smear (ZN)	140	112	168	Gamma	11
	Culture (MGIT)	3960	3168	4752	Gamma	11
	ECG	1495	1196	1794	Gamma	5
	Liver function test	2813	2250	3376	Gamma	5
	Complete blood count	1483	1186	1780	Gamma	5
	Thyroid-stimulating hormone	752	602	902	Gamma	5
	Staff incentives	5000	4000	6000	Gamma	4
	Patient incentives (NPY)	9500	7600	11,400	Gamma	13
	Travel—patient	6000	4800	7200	Gamma	14
	Hospitalization for ADR	4945	3956	5934	Gamma	14

NA = not applicable; TB = tuberculosis; ADR = adverse drug reaction; INR = Indian rupees.

**Table 4:** Incremental cost-effectiveness of BEAT-TB when compared to 18-month shortened pre-XDR TB treatment regimen.

Strategy	Total for 1000-Patient Cohort			Incremental			ICER	
	Cost (INR)	Life Years	QALY	Cost (INR)	Life Years	QALY	Life Years	QALY
6-month regimen (BEAT-TB)	64,866,321	40,548	27,633	−10,609,875	19,539	11,821	−543	−898
Comparator								
18-month regimen	75,476,196	21,009	15,812	-	-	-	-	-

QALY = quality-adjusted life year.

## Data Availability

All data generated during this study are included in this published article.
